# Evaluation of the effects of a VEGFR-2 inhibitor compound on alanine aminotransferase gene expression and enzymatic activity in the rat liver

**DOI:** 10.1186/1476-5926-10-8

**Published:** 2011-08-17

**Authors:** Carmen Fuentealba, Monali Bera, Bart Jessen, Fred Sace, Greg J Stevens, Dusko Trajkovic, Amy H Yang, Winston Evering

**Affiliations:** 1 Drug Safety Research & Development, Pfizer Inc., La Jolla, CA, Michigan State University, USA; 2 Faculty of Veterinary Medicine, University of Calgary, HRIC 2C56, 3330 Hospital Drive NW, Calgary, AB T2N 4N1, Canada

**Keywords:** Drug safety, hepatotoxicity, liver enzymes, ALT gene expression

## Abstract

**Background:**

Traditional assessment of drug-induced hepatotoxicity includes morphological examination of the liver and evaluation of liver enzyme activity in serum. The objective of the study was to determine the origin of drug-related elevation in serum alanine aminotransferase (ALT) activity in the absence of morphologic changes in the liver by utilizing molecular and immunohistochemical techniques.

**Methods:**

Sixteen female Sprague-Dawley rats were divided into 2 groups (control and treated, n = 4 per group) and treated rats were dosed orally twice daily (400 mg/kg/day) for 7 days with a VEGFR-2 compound (AG28262), which in a previous study caused ALT elevation without morphological changes. Serum of both treated and control animals were evaluated on day 3 of treatment and at day 8. Three separate liver lobes (caudate, right medial, and left lateral) were examined for determination of ALT tissue activity, ALT gene expression and morphological changes.

**Results:**

ALT activity was significantly (p < 0.01) elevated on day 3 and further increased on day 8. Histologic changes or increase in TUNEL and caspase3 positive cells were not observed in the liver lobes examined. ALT gene expression in the caudate lobe was significantly up-regulated by 63%. ALT expression in the left lateral lobe was not significantly affected. Statistically significant increased liver ALT enzymatic activity occurred in the caudate (96%) and right medial (41%) lobes but not in the left lateral lobe.

**Conclusions:**

AG28262, a VEFG-r2 inhibitor, causes an increase in serum ALT, due in part to both gene up-regulation. Differences between liver lobes may be attributable to differential distribution of blood from portal circulation. Incorporation of molecular data, such as gene and protein expression, and sampling multiple liver lobes may shed mechanistic insight to the evaluation of hepatotoxicity.

## Background

The liver provides many essential functions such as regulation of amino acids and glucose in the blood, production of bile, and the biotransformation of toxins and drugs. The liver is the first organ to encounter nutrients, drugs and toxins absorbed into the enteric system through the portal vein [1]. Many of the toxins, which pass through the liver are metabolized and excreted using numerous metabolic pathways involving specialized enzymes specifically for detoxification. Because of the liver's important role in biotransformation of drugs and toxins, drug-induced hepatotoxicity is a major concern in drug development and chronic drug therapy.

A common, liver specific biomarker used to evaluate acute hepatotoxicity is Alanine aminotransferase (ALT). ALT is a cytosolic enzyme found in hepatocytes, and is frequently examined in patients undergoing chronic drug therapy or in the pre-clinical stages of drug development to monitor the status of the liver. Serum concentrations of ALT rise in response to direct damage to hepatocytes or through leakage resulting from altered cell metabolism [2]. ALT is commonly evaluated in conjunction with aspartate aminotransferase (AST), a nonspecific enzyme found in the liver, as well as histologic morphology of the liver [3]. Drug related discrepancies have been identified where elevation in serum ALT is detected without a hepatic morphologic correlation. An example of this includes isoniazid, a compound that induces an elevation in serum ALT and AST activity without directly causing hepatic damage [3]. Another example, diclofenac, a non-steroidal anti-inflammatory drug also has been reported to elevate serum aminotransferase activity; however some patients progressed to consequentially develop liver disease [4].

Elucidating the drug-related mechanism which elevates serum ALT activity is crucial to better understand the potential for consequent hepatic disease. This study investigates potential mechanisms resulting in elevated serum ALT activity using rats treated with a VEGFR-2 inhibitor (AG28262). Vascular endothelial growth factor (VEGF) induces angiogenesis and is a potent mediator of vascular permeability. The biological effects of VEGF are mediated by two tyrosine kinase receptors, Flt-1 (VEGFR-1) and KDR (VEGFR-2). Inhibition of VEGF activity may be beneficial in the treatment of conditions involving angiogenesis [5]. Since the liver is a heterogeneous tissue and lobe variation has been reported in hepatotoxicity [6], three liver lobes (caudate, right medial and left lateral) were selected for examination using morphological evaluation and molecular techniques.

## Methods

### Animals

Eight female Sprague-Dawley rats (Charles River Laboratories, Raleigh, NC) weighing between 220-250 grams were used in the study. Animals were allowed to acclimate for one-week prior to use. All animals were given food and water ad-libitum, and housed under a 12-hour light/12-hour dark cycle.

### Institutional compliance statement

Animals were housed in facilities at Pfizer (La Jolla, CA, USA) that are approved by the American Association for the Accreditation of Laboratory Animal Care. All protocols were approved by the Pfizer Global Research and Development Institutional Animal Care and Use Committee.

### Study design

Animals were assigned to a control (0.5% carboxymethylcellulose) and a treated group (400 mg/kg/day of AG028262) and were dosed orally twice daily for seven consecutive days (n = 4 per group). Clinical observation was performed daily. Body weights were taken on days 1, 6, and 8. Clinical chemistry and hematological samples were collected on day 8 via blood collection from the abdominal vena cava. In addition, clinical chemistry was evaluated on day 3 during treatment via tail vein collection. ALT, ALP and AST enzymatic activity and other biochemical tests were performed with a Hitachi 911 chemical analyzer using a standardized method. A necropsy was conducted on each rat on day 8 and gross observations were recorded. The left lateral, right medial and caudate lobes of the liver were collected, weighed, and examined for gross lesions. Liver lobes were selected based upon the differential distribution of the portal hemodynamics through the liver lobes [7]. Tissue for RNA analysis was collected in RNA later (Qiagen, Valencia, CA) and directly transferred to liquid nitrogen. Tissue for protein quantification was directly transferred to liquid nitrogen for freezing. The remaining tissues were fixed in 10% neutral buffered formalin and submitted to histology for processing and staining with H&E, caspase 3 and TUNEL method.

### RNA isolation and reverse transcription

Tissues were homogenized by an ultra turrax homogenizer (IKA Works, Wilmington, NC) and RNA extracted using the RNeasy Lipid tissue midi kit) (Qiagen). Oligo-dT primed reverse transcription was carried out with 1 μg total RNA using the Retroscript kit (Ambion; Austin, TX). For detecting gene expression of alanine aminotransferase (ALT), the following primers were used: 5'-TTCAAGCAGAGAGACAGGAG-3' and 5'-TGAGGGAAGGAATACATGG-3.' The primers for β-actin, used as a reference gene to normalize expression levels between samples, were: 5'- CTCACTGTCCACCTTCCAG-3' and 5'- AACGCAGCTCAGTAACAGTC-3.' To amplify and quantitate cDNA, 1 μl of cDNA generated by reverse transcription was added to 19 μl of PCR mix containing SYBER green PCR master mix (Qiagen), 2 μM primers, and RNAse free water. The reaction was performed by Light Cycler (Roche Diagnostics, Indianapolis IN). PCR cycle settings for ALT were set 94°C for 15 s, followed by 52°C for 20 s, and 72°C for 30 s for 50 cycles. For β-actin reactions the annealing temperature was changed to 55°C. Light Cycler software version 3.5 (Roche) was used for data analysis. Standards generated from traditional PCR reactions were included in each amplification run to generate a standard curve off of which samples were quantified and expressed as a relative value. Values were then normalized to the reference gene to generate gene expression results expressed as a relative ratio.

### Cleaved caspase 3 and TUNEL

Samples of the caudate, right medial, and left lateral liver lobes were paraffin-embedded, serially sectioned at 4 μm, mounted onto positively charge plus slides (VWR) and stained for markers of apoptosis. Deparaffinization and antigen retrieval were performed in 1X Reveal solution using a Decloaking Chamber (Biocare Medical, Walnut Creek, CA). Endogenous peroxidase activity was blocked using 3% hydrogen peroxide (Sigma, St. Louis, MO). The Dako Autostainer (Dako-Cytomation, Carpinteria, CA) was programmed to complete the immunohistochemistry staining for caspase 3. Protein Blocking Serum (Dako) was used first to reduce background staining. Caspase-3 polyclonal antibody (1:200 dilution; Cell Signaling, Beverly, MA) was the primary antibody directed against cleaved caspase-3. The negative control consisted of replacing the primary antibody with non-specific Rabbit IgG antibody (Dako). Biotinylated anti-rabbit immunoglobulin (1:200 diluted in Dako Antibody Diluent) was used as the secondary antibody. Antibody binding was visualized using streptavidin peroxidase (1:200 diluted in antibody diluent) and DAB+ chromogen followed by hematoxylin counterstain. Terminal deoxynucleotidyl transferase (Tdt)-mediated dUTP nick-end labeling (TUNEL) was performed using the DeadEnd Colorimetric TUNEL system (Promega, Madison, WI). Briefly, sections were rehydrated in decreasing concentration of ethanol followed by a wash in 0.85% NaCl (Sigma) for 5 minutes. After a final wash in PBS, sections were fixed in 10% formalin in PBS (Richard Allen Scientific, Kalamazoo, MI) for 15 minutes. To help permeabilize tissue, sections were incubated in Proteinase K (Dako) for 20 minutes. The remaining steps including equilibration and end labeling reaction were followed per manufacturer's protocol (Promega). Apoptotic cells were detected after incubation in DAB chromogen (Invitrogen; Carlsbad, CA) for 2.5 minutes followed with hematoxylin counterstaining (Dako). All slides were cover slipped using permanent mounting medium (Richard Allen Scientific).

### Crude liver ALT quantification

Liver tissue (50 mg of each lobe) was weighed and homogenized using the ultra turrax homogenizer in 1 mL buffer (100 mM phosphate buffer at pH 7.4, 0.25 M Sucrose, 0.01 mM EDTA), complete protease inhibitor cocktail tablets (Roche), and 2 mM PMSF. Samples were centrifuged at 2500 g, 4°C for 15 minutes. ALT enzymatic activity in the supernatant was quantified (U/L) using the Hitachi 911 Analyzer (Roche) at 37°C. Pig heart ALT (Roche) of known enzymatic activity was used to verify the performance of the Hitachi 911 in measuring enzymatic activity in crude tissue.

### Morphometry

Chromavision (Chromavision Medical Systems, San Juan Capistrano, CA) was utilized for morphometrical analysis of caspase 3 and TUNEL stained slides. Quantification was done by a pre-programmed logarithm directed specifically in the identification of caspase 3 and TUNEL stained slides.

### Statistical analysis

Statistical analysis was done by 2-sample equal variance t-Test. Significance was set at p ≤ 0.01.

## Results

### In-vivo observations

Significant changes in body weights and clinical signs were not observed for the 7-day duration of the study. There were no unscheduled deaths in the study or significant changes found during gross examination. Differences in liver weight between controls and treated animals were not observed.

### Histology

No significant morphologic changes were observed in the livers of compound-treated or control rats (data not shown). Differences between liver lobes were not detected.

### Caspase 3 and TUNEL

Few caspase 3 and TUNEL positive cells were seen in the livers of both treated and control rats. No significant statistical differences between these groups were detected using morphometry.

### Clinical pathology

ALT, ALP, and AST activity was measured in the serum of compound-treated and control rats and results are presented in Table 1. On day 3 of treatment, AG28262 induced a statistically significant increase in serum ALT activity (63%; p ≤ 0.01) compared to controls. On day 8, ALT activity progressively increased by approximately 2-fold compared to the control group, a statistically significant difference (p ≤ 0.01). There was a progressive increase in serum ALP activity from day 3 to day 8 in treated animals, and the increase in treated rats at day 8 was statistically significant compared to controls. Serum AST activity in treated rats was increased by 63% on day 8 compared to the control rats but the increase was not statistically significant due to individual variability.

**Table 1:** Effect of AG28262, a VEGR-2 inhibitor, on serum ALT, ALP and AST enzymatic activity in treated and control rats

Group	Day sampled	ALT (U/L)	ALP (U/L)	AST (U/L)
Control	3	53 ± 2	175 ± 15	99 ± 3
400 mg/kg	3	82 ± 7 *	197 ± 16	111 ± 1
Control	8	55 ± 4	153 ± 12	89 ± 2
400 mg/kg	8	118 ± 19*	209 ± 15*	150 ± 40

Values expressed as mean U/L ± SEM.

*Statistically significant (p ≤ 0.01).

### AG28262-induced effect on ALT gene expression

**Figure 1:**
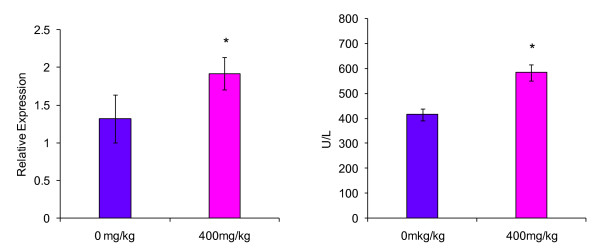
**Effect of AG28262, a VEGR-2 inhibitor, on ALT gene expression and enzymatic activity in the right medial liver lobe**. Relative gene expression values are reported as mRNA ALT/mRNA beta-actin. * Statistically significant (p < 0.01).

**Figure 2:**
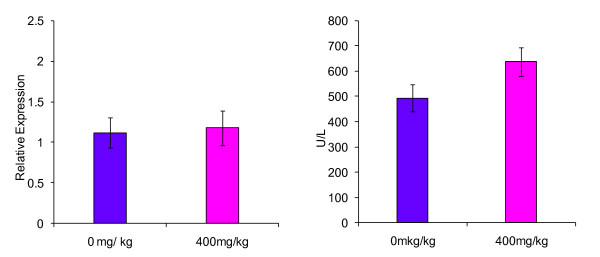
**Effect of AG28262, a VEGR-2 inhibitor, on ALT gene expression and enzymatic activity in the left lateral liver lobe**. Relative gene expression values are reported as mRNA ALT/mRNA beta-actin.

**Figure 3:**
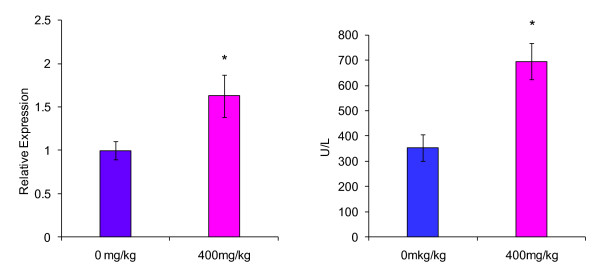
**Effect of AG28262, a VEGR-2 inhibitor, on ALT gene expression and enzymatic activity in the caudate liver lobe**. Relative gene expression values are reported as mRNA ALT/mRNA beta-actin.

In the right medial lobe, AG28262 treatment resulted in a 49% increase ALT gene expression compared to the control animals on day 8 (Figure 1). Relative expression of ALT in the left lateral liver lobe at day 8 of termination was not significantly different from the control group (Figure 2). The caudate lobe had a statistically significant (p ≤ 0.01) increase in ALT gene expression of 63% in comparison to the control group (Figure 3).

### AG28262-induced effect on crude liver ALT enzymatic activity

Both the right medial and caudate lobes demonstrated a statistical increase in ALT enzymatic activity when compared to the control with 41% (p ≤ 0.01) and 96% (p ≤ 0.01) increase respectively (Figures 1 and 3). Enzymatic ALT activity in the left lateral lobe was elevated by 29% in comparison to the control (Figure 2), but the difference was not statistically significant.

## Discussion

Differences in drug effects between liver lobes should be considered in toxicology evaluation of compounds. Traditional thinking regarding drug-induced hepatotoxicity commonly correlates elevated serum ALT with direct hepatocellular damage. However, instances of elevated serum ALT in the absence of microscopic evidence of hepatocellular injury do occur with some xenobiotics. This investigation was conducted to understand the ALT elevation observed with AG28262, a VEGFR-2 inhibitor, in treated rats in the absence of morphological changes in the liver. The results of this investigation suggests that the source of increased serum ALT in AG28262 treated rats is due to an increase in gene expression rather than leakage as a result of overt hepatocellular necrosis. This study also showed a regional specific effect on ALT mRNA and protein levels within the various lobes of the liver.

In an effort to rule out drug-induced hepatocellular apoptosis as a potential cause of increases in serum ALT activity, caspase 3 immunohistochemistry and TUNEL assays were used. Both assays demonstrated equivalent positive staining in the compound-treated and control rats. This information suggests that elevation in serum ALT was not due to hepatocellular apoptosis, but to an alternative mechanism. The results obtained from caspase 3 and TUNEL assays further supported the lack of morphologic hepatic changes.

AG28262 treatment resulted in increased activity of ALT, AST, and ALP suggesting that AG28262 induces hepatic injury. Clinical chemistry data demonstrated a statistically significant increase in serum ALT, ALP activities, and increased (but not statistically significant) AST activity on day 8. Serum AST activity on day 8 showed individual variability within the compound-treated group; however there was still a remarkable elevation when compared to control animals. ALT, AST, and ALP are all enzymes found in the liver and are commonly used in conjunction to evaluate hepatic changes [8]. Despite these elevations in liver enzyme activity there were not morphological correlates within the liver.

Muscle and kidney are two other sources of ALT that may contribute to the elevation in serum ALT in this study. However, creatine kinase serum enzymatic activity, a specific marker for muscle or kidney damage [9-12], was not significantly changed in this study. Morphological changes were not observed in these tissues and further studies were not pursued at the time.

Real time PCR was used to measure changes in ALT gene expression between the treated and control animals. Using beta-actin for normalization, AG28262 elicited an increased in hepatic ALT mRNA levels. Additionally, regional differences among the lobes of the liver were observed in AG28262 treated rats. The largest increase in ALT mRNA was in the caudate lobe, followed by the right medial, and lastly the left lateral lobe. The caudate lobe showed a 63% significant increase in gene expression comparison to the control. Gene expression in the treated right medial lobe was also increased by 49%; however, individual variability within the group prevented the result from reaching statistical significance. AG28262 induced a slight change in gene expression in the left lateral lobe.

A correlation between crude liver ALT enzymatic activity in the lobes and ALT gene expression was identified. The caudate lobe, which had significant elevations in gene expression, also demonstrated a significant elevation in ALT enzymatic activity. The right medial lobe also showed a significant increase in ALT enzymatic activity, which correlated with elevation in ALT gene expression. The left lateral lobe had a slight increase in ALT concentration, which may be due to only a minor increase in gene expression. These data suggest that the effect of AG28262 is targeted towards ALT gene regulation resulting in increased synthesis of ALT enzyme in the hepatocytes. The source of serum ALT appears to originate from the liver, but more specifically the caudate and right medial liver lobes.

The variability on ALT activity between the liver lobes confirms the heterogeneity of the liver and warrants the investigation of multiple liver lobes in future drug toxicity studies. Previous hepatotoxicity studies involving copper and acetaminophen have supported the idea of lobular heterogeneity [13,14]. Both copper and acetominophen have been studied extensively and it has been shown that effect of both toxins is differential in nature. The distributional effect of copper, for example is thought to reflect the site of gastrointestinal absorption and portal streamlining into the liver [14]. Other studies have indicated that the right liver lobe is predisposed to the effects of drugs and toxins based on favored portal streamlining to the right portal branch which supplies the right side of the liver [6]. The effects of AG28262 in this study were clearly concentrated in the right medial and caudate liver lobes suggesting that the compound may preferentially be transported through the right portal branch into the right side of the liver. The caudate lobe of the liver has been previously shown to receive blood supply from the right and left branches of the portal vein [7]. Insight on the potential distribution of drugs and toxins may help in understanding the potential localization of hepatic diseases and carcinomas within the liver. Understanding these regional effects is critical in the interpretation of data that captures endpoints from specific liver lobes (eg. toxicogenomics).

The combination of ALT gene up-regulation and a lack of morphologic change support the importance of utilizing toxicogenomics in evaluating potential drug related changes. Toxicogenomics is a relatively new tool incorporating genomics and proteomics and can prove useful in short-term drug toxicity studies because gene and protein changes can be detected before drug induced morphologic changes [15]. A study involving acetaminophen toxicity demonstrated that gene expression profiling serves as an important indicator of potential toxic effects in the absence of apparent toxicity [16]. Collection of samples for gene expression analysis is not done routinely in exploratory toxicology studies. Such practice may prove useful so that the mechanisms of findings such as those reported in this study can be explored. In this study genomics proved useful in identifying the cause and source of serum ALT elevation. It is still unknown whether the chronic effect of AG28262 will result in morphologic changes or if the compound will independently alter the intrinsic regulation of ALT gene expression and synthesis. Further investigation is necessary to determine if effects of the compound are occurring ultrastructurally, biochemically, or if there is involvement of a transcription factor, which may be altering gene expression.

## Competing interests

The authors declare that they have no competing interests.

## Authors' contributions

CF, GJS and WE have made substantial contributions to conception and design of the study, MB performed the experiments during a research rotation (part of her DVM program), FS carried out the clinical pathology tests and implemented the techniques for detection of liver enzymes in tissues, DT carried out the histology and implemented the immunohistochemical techniques, BJ assisted in implementation of toxicogenomics and interpreting data and AHY contributed to carry out toxicogenomics. CF coordinated the study and drafted the manuscript. All authors read and approved the manuscript content.
